# Evolution of the endomembrane systems of trypanosomatids – conservation and specialisation

**DOI:** 10.1242/jcs.197640

**Published:** 2017-04-15

**Authors:** Divya Venkatesh, Cordula Boehm, Lael D. Barlow, Nerissa N. Nankissoor, Amanda O'Reilly, Steven Kelly, Joel B. Dacks, Mark C. Field

**Affiliations:** 1 Wellcome Trust Centre for Anti-Infectives Research, School of Life Sciences, University of Dundee, Dow Street, Dundee DD1 5EH, UK; 2 Department of Pathology, University of Cambridge, Tennis Court Road, Cambridge CB2 1PQ, UK; 3 Department of Cell Biology, University of Alberta, Edmonton, Alberta T6G 2H7, Canada; 4 Department of Plant Sciences, University of Oxford, Oxford OX1 6JP, UK

**Keywords:** Rab, SNARE, Trafficking, Molecular evolution, TBC domain, Trypanosoma, Proteomics

## Abstract

Parasite surfaces support multiple functions required for survival within their hosts, and maintenance and functionality of the surface depends on membrane trafficking. To understand the evolutionary history of trypanosomatid trafficking, where multiple lifestyles and mechanisms of host interactions are known, we examined protein families central to defining intracellular compartments and mediating transport, namely Rabs, SNAREs and RabGAPs, across all available Euglenozoa genomes. Bodonids possess a large trafficking repertoire, which is mainly retained by the *Trypanosoma cruzi* group, with extensive losses in other lineages, particularly African trypanosomes and phytomonads. There are no large-scale expansions or contractions from an inferred ancestor, excluding direct associations between parasitism or host range. However, we observe stepwise secondary losses within Rab and SNARE cohorts (but not RabGAPs). Major changes are associated with endosomal and late exocytic pathways, consistent with the diversity in surface proteomes between trypanosomatids and mechanisms of interaction with the host. Along with the conserved core family proteins, several lineage-specific members of the Rab (but not SNARE) family were found. Significantly, testing predictions of SNARE complex composition by proteomics confirms generalised retention of function across eukaryotes.

## Abstract (precis)

**Summary:** The trafficking systems of trypanosomes are shown to vary considerably, with much minimisation in systems with antigenic variation and more complexity in cell invasive forms.

## INTRODUCTION

Membrane trafficking mediates delivery of macromolecules to discrete intracellular compartments from the site of uptake or synthesis. Trafficking is essential to nearly all eukaryotic cells, contributing towards nutrient acquisition, protein processing and turnover, and, in multicellular organisms, it also participates in higher-order tissue organisation. The importance of membrane transport is reflected in the many diseases associated with trafficking, including diabetes, Alzheimer's disease and cystic fibrosis (Birault et al., 2013; Olkkonen and Ikonen, 2006; Rajendran and Annaert, 2012; Seixas et al., 2013). Development of membrane trafficking was likely a major evolutionary driver enabling the transition from prokaryotic to eukaryotic cells (Field et al., 2011; Dacks et al., 2016). For many pathogens, trafficking has special relevance in maintaining the host–parasite interface, which is at the cell surface, and both the surface and underlying trafficking apparatus are intimately connected with immune evasion, pathogenesis and life cycle progression (Manna et al., 2013).

Membrane transport requires vesicle formation, translocation, tethering, docking and fusion to release cargo (Bonifacino and Glick, 2004), which is achieved though coordinated action by Rab and ARF GTPases, coat complexes, tethers and SNAREs. Members of these paralogous families encode specificity for individual transport events to defined organelles; evolutionary reconstructions suggest stepwise evolution from a simpler ancestral system before the last eukaryotic common ancestor (LECA) (Devos et al., 2004, 2006; Dokudovskaya et al., 2006; Field and Dacks, 2009; Schlacht et al., 2014). A conserved core of Rab, RabGAP and SNARE proteins has been derived by reconstructing eukaryotic evolutionary history and broadly supports this model (Arasaki et al., 2015; Diekmann et al., 2011; Elias et al., 2012; Kienle et al., 2009a; Vedovato et al., 2009; Yoshizawa et al., 2006). Beyond this are examples of lineage-specific features in the endosomal sorting complexes required for transport (ESCRT) system, sortillins and ARF GTPases in metazoans (Field et al., 2007; Gabernet-Castello et al., 2013; Leung et al., 2008). In parasitic organisms, adaptin complexes, important cargo selectors that are otherwise well conserved in most lineages, are rather variable, possibly because of specific adaptation (Woo et al., 2015).

Kinetoplastids are unicellular flagellated protists within the supergroup Excavata, and include free-living species and parasites that cause many important human diseases, together with species afflicting animals and plants. Kinetoplastids exhibit varied lifestyles, host range, distribution and specialisations over long evolutionary periods. Furthermore, kinetoplastid surfaces are highly divergent between lineages, likely reflecting specialisations within membrane transport (Gadelha et al., 2015; Manna et al., 2013). More remarkable is the highly distinct nature of the proteins and glycoconjugates present at the cell surface, with recent data indicating that many surface proteins are restricted to the kinetoplastids (Adung'a et al., 2013; Field et al., 2007; Jackson, 2016; Gadelha et al., 2015). All of these features can be anticipated as leaving an imprint in the evolutionary history of the trafficking system. To address how kinetoplastid trafficking pathways evolved, we examined the representation of Rabs, RabGAPs and SNAREs across all currently available genome/transcriptome resources.

SNAREs, mediators of membrane fusion, possess a characteristic domain, and are classified as Q- or R-SNAREs (Bock et al., 2001; Fasshauer et al., 1998). Typically, three Q- (a, b or c) and one R-SNARE form a complex. The complement of Qa and R-SNAREs in the LECA has been established with broad taxonomic sampling (Arasaki et al., 2015; Vedovato et al., 2009), but the Qb and Qc families were only assessed by comparative genomics nearly a decade ago, using the limited sampling of genomes then available (Kloepper et al., 2007; Yoshizawa et al., 2006) and warrant re-examination to solidify the definition of the LECA complement. It is key to have a robust estimate of the overall SNARE complement in the LECA as the starting point from which we assume kinetoplastids evolved. Rabs are small GTPases and well established markers of organellar identity (Brighouse et al., 2010; Pereira-Leal and Seabra, 2001). A broad repertoire of ∼23 Rabs are predicted in the LECA (Elias et al., 2012). With the exception of metazoa and vascular plants, where great expansion is evident, Rab proteins have evolved mainly through small scale expansions and secondary losses, plus emergence of novel paralogs (Elias et al., 2012; Klöpper et al., 2012). Rabs are regulated by GTPase-activating proteins (GAPs), the majority of which possess a Tre-2/Bub2/Cdc16 (TBC) Rab-binding domain. The LECA possessed about ten TBC subtypes and subsequent expansions have included domain swapping (Gabernet-Castello et al., 2013). The greatest number of TBC innovations are in animals and fungi, although novel subclasses are present in a wide range of lineages. Detailed analysis of both Rab and SNARE repertoires in fungi indicates a simple set with minimal variations between different lineages, despite major diversity in morphology and transitions between single and multicellular forms, albeit with evidence for minimisation in some species (Kienle et al., 2009b; Pereira-Leal, 2008).

Our study indicates that a significant proportion of the putative LECA repertoire of these protein families is conserved in kinetoplastids, along with some lineage-specific proteins and secondary losses. We infer a large ancestral repertoire of Rab and SNARE proteins (but not TBC Rab-GAPs) associated with endosomal and late exocytic pathways that appears to undergo step-wise secondary loss in several parasitic trypanosomatid lineages (but not so in the *Trypanosoma cruzi* group). The proteins associated with these pathways also show high levels of variation in numbers and level of conservation within the kinetoplastids, even though no large-scale changes obviously correlate with parasitism, host range or modes of immune evasion.

## RESULTS

### Confirmation of the LECA complement of Qb and Qc SNAREs

**Fig. 1.**
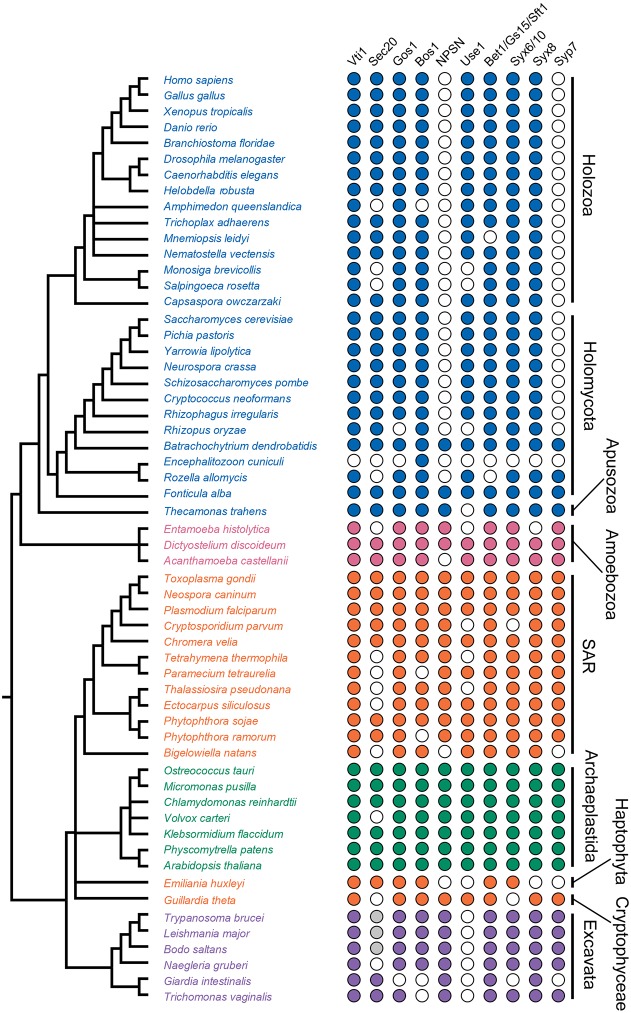
**Distribution of Qb and Qc SNARE family members across a diverse sampling of eukaryotes.** Colour-filled circles indicate the presence of at least one identified orthologue, while empty circles indicate failure to identify any orthologues. Grey-filled circles indicate identification in downstream analyses focused specifically on kinetoplastid genomes (see Fig. 5A). Identified sequences and sources are listed in Tables S1, S3 and S4.

Although recent analyses have addressed most protein families and subfamilies considered here, Qb and Qc SNAREs have not been characterised in a comparable way. In particular, there are conflicting views of the distributions of Qb-SNARE Novel Plant Syntaxin (NPSN) and the Qc-SNARE Syntaxin of Plants 7 (Syp7) (Sanderfoot et al., 2000). As their names imply, both were proposed as being plant specific, but other studies suggest orthologues in several protist lineages, including *Trypanosoma* and *Dictyostelium* (Kloepper et al., 2007; Sanderfoot, 2007; Yoshizawa et al., 2006), which prompted some authors to suggest that NPSN, at least, was present in the LECA (Kienle et al., 2009a). To determine whether NPSN and Syp7 orthologues are present in non-plant eukaryotes, we performed comparative genomics and phylogenetic analysis. Searches returned NSPN and Syp7 candidate orthologues from the genomes of most eukaryotes except animals and non-basal fungi (Fig. 1). A Qb-SNARE phylogeny strongly supports the identity of these putative NPSN orthologues and, similarly, the Qc-SNARE tree strongly supports identity of Syp7 orthologues (Fig. S2F,G). This analysis suggests that both NPSN and Syp7 were present in the LECA and later lost from animals and fungi.

### The kinetoplastid complement of trafficking proteins

**Fig. 2.**
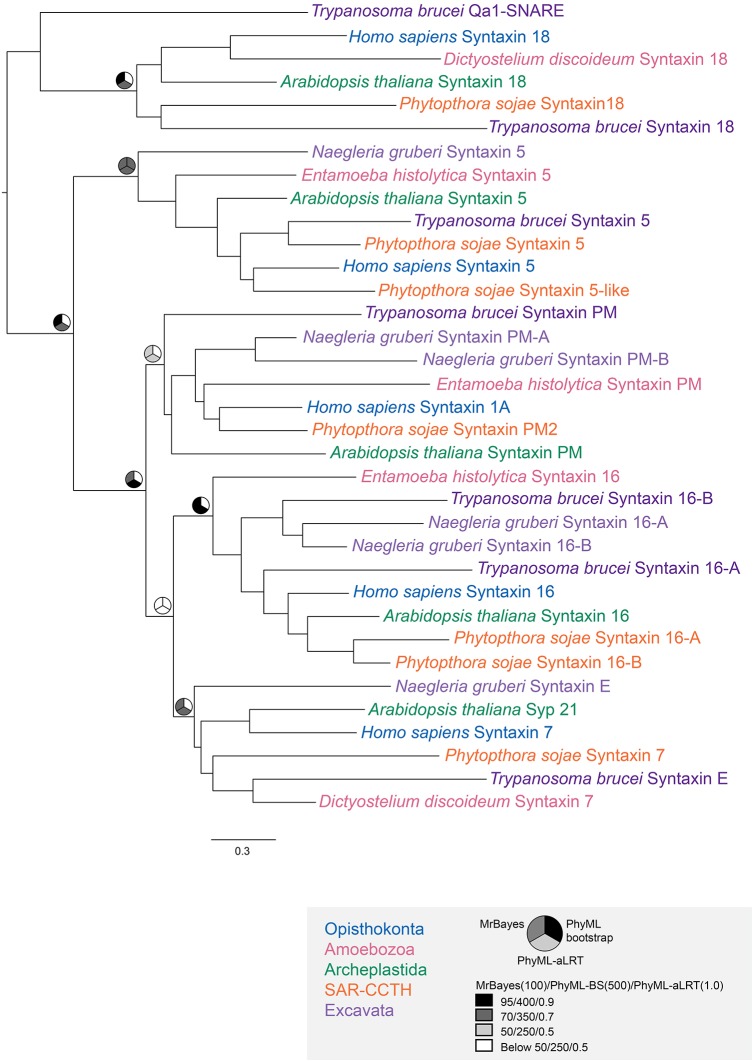
**Phylogenetic assignment of kinetoplastid Qa SNAREs.** The optimum PhyML topology is presented. Node values are iconised as pie charts for three support values, representing PhyML approximate likelihood ratio test, PhyML Bootstrap and MrBayes posterior probabilities and colour-coded as indicated. Each phylogeny shows one representative kinetoplastid SNARE from each sub-type (purple) along with eukaryotic representative SNAREs from Opisthokonta (blue), Amoebozoa (pink), Archaeplastids (green), SAR-CCTH (orange) and Excavata (light purple). Qa SNAREs showing orthology with eukaryotic orthologues for Syx18, Syx5, Syx16, SynE and SynPM.

**Fig. 3.**
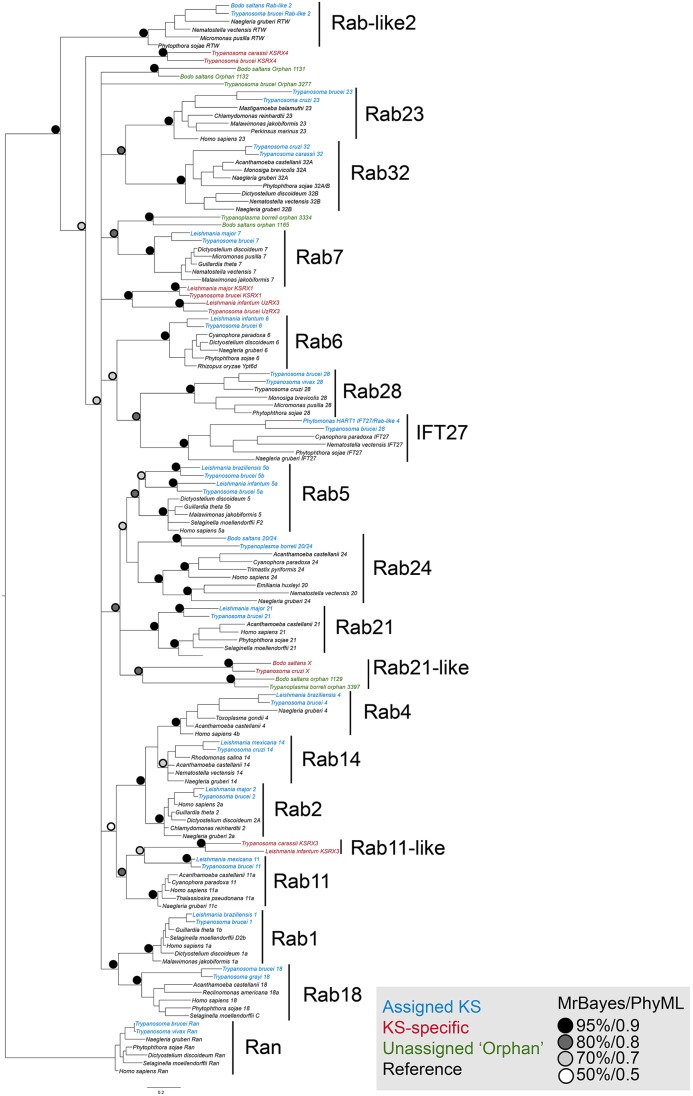
**Phylogenetic relationships of kinetoplastid Rabs.** The consensus Bayesian topology is shown. Nodes are iconised as colour-coded circles according to MrBayes posterior probabilities and PhyML approximate likelihood ratio test as shown in the key. The figure includes two representative kinetoplastid Rabs from each subfamily cluster along with representative Rabs from across eukaryotes. Assigned kinetoplastid-specific Rabs are in blue, lineage-specific Rabs in red, unassigned orphan Rabs in green, while representative eukaryotic Rabs are black. LECA Rabs for which no kinetoplastid orthologues were found (Rabs 8, 22, 34, 50 and Titan) were not included in the analysis. Ran is used as outgroup.

**Fig. 4.**
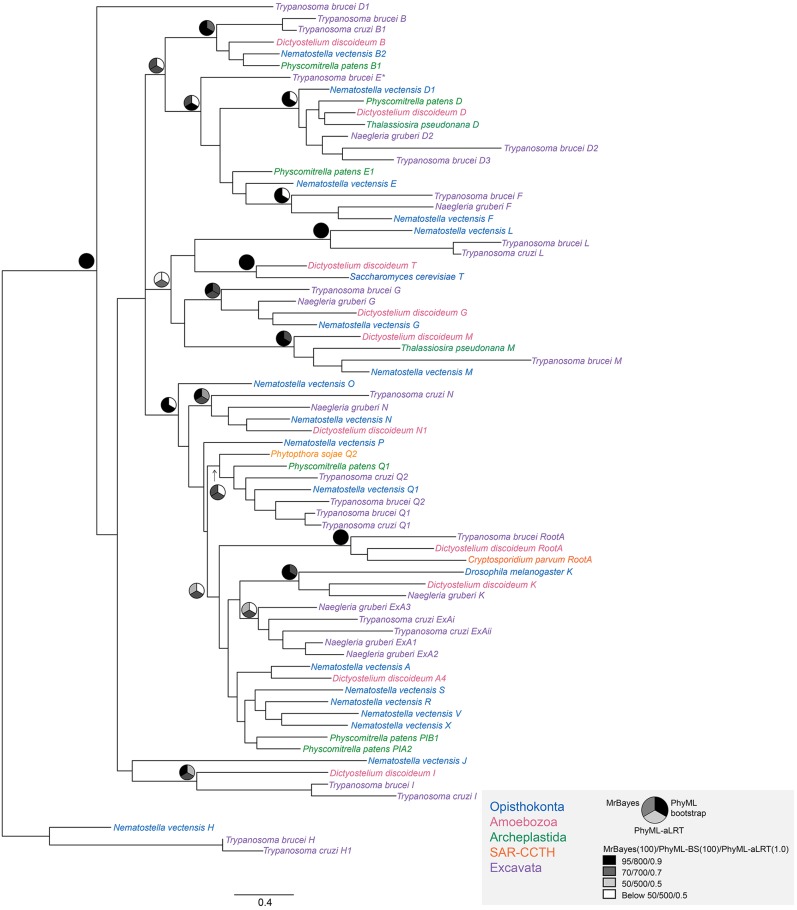
**Phylogenetic relationships of kinetoplastid TBC-domain-containing predicted proteins.** The optimum PhyML topology is shown. Node values are iconised as pie charts as in Fig. 4. Each phylogeny shows one representative kinetoplastid TBC from each sub-type cluster (purple) along with eukaryotic representative SNAREs from Opisthokonta (blue), Amoebozoa (pink), Archaeplastida (green), SAR-CCTH (orange) and Excavata (light Purple). TBCs B, D, F, L, M and Root A are supported by at least two support values above 90/90/0.9 (for MrBayes/PhyML-BS/PhyML-aLRT, respectively) and TBCs G, N, K and I have at least one value above 0.9/90/90 confidence. TBC-Q has two support values above 0.7/70/70, while TBC-ExA has even lower support with one value over 0.5/50/50 and one over 0.7/70/70. In the latter case this suggests that the clade may not be monophyletic. The protein previously described as TBC-E, failed to resolve and is highlighted with an asterisk.

We next considered the genomes of 18 Euglenozoa including basal bodonids and trypanosomatids. We identified candidate genes for 382 Rabs, 552 SNAREs and 307 TBC RabGAPs. Where possible these were assigned to subfamilies by phylogenetic analyses with a reference set of previously defined sequences from across eukaryotes. Such data are readily available for Rabs and TBC RabGAPs (Elias et al., 2012; Gabernet-Castello et al., 2013) and were prepared here for SNAREs using SNARE repertoires from at least one representative of each eukaryotic subgroup, other than the Excavata; *Homo sapiens* and *Saccharomyces cerevisiae* as Opisthokonta, *Dictyostelium discoideum* as Amoebozoa, *Phytopthora sojae* as SAR-CCTH (a heterogenous group comprising stramenopiles, alveolates and Rhizaria along with cryptomonads, centrohelids, telonemids and haptophytes) and *Arabidopsis thaliana* for the Archaeplastida. We were able to assign a considerable proportion of the kinetoplastid representatives of all three families (Fig. 2, 3, 4; Fig. S2). Moreover, 86% of LECA SNAREs, 72% of LECA Rabs and 100% of LECA TBC RabGAPs were identified in one or more members of the lineage, suggesting that the core machinery of trafficking is well conserved (Table 1). Most kinetoplastid proteins were assigned by phylogenetic evidence, but three SNARE proteins, VTI1-like, Syx6-like and Syx8-like, were assigned only by BLAST and reverse BLAST as phylogenetic support was low.

**Table 1.**
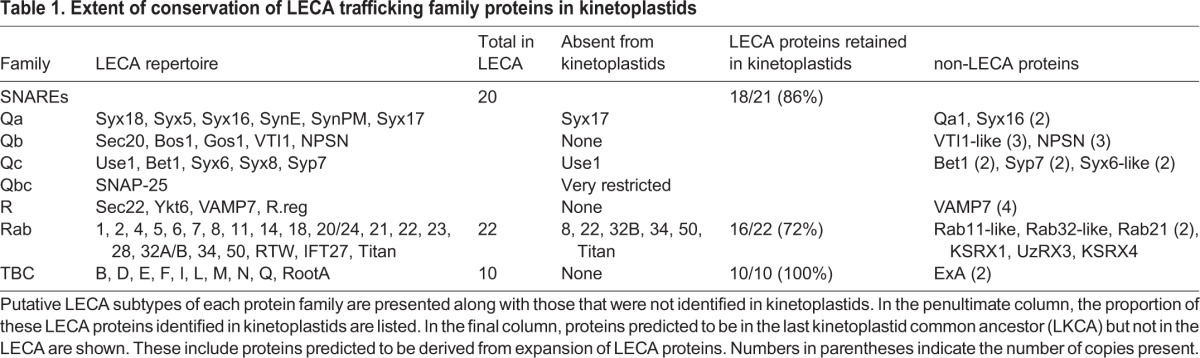
**Extent of conservation of LECA trafficking family proteins in kinetoplastids**

### Comparing coding content and trafficking repertoire

There is loose correspondence between genome and Rab repertoire sizes (Gabernet-Castello et al., 2013). While this may reflect a connection between genome and compartmental complexity, the huge diversity within eukaryotes makes complexity a difficult parameter to define, and analysing a group of organisms where the basic cellular bauplan is well conserved, allows us to re-evaluate this concept (Fig. S1). The coding content of *Bodo* *saltans* is considerably larger than the parasites, while the excavates *Naegleria* *gruberi* and *Euglena* *gracilis* have larger genomes (Ebenezer et al., 2017; Fritz-Laylin et al., 2010). In keeping with genome size, *B. saltans* and *N. gruberi* possess greater numbers of SNAREs, Rabs and TBCs compared to kinetoplastids. Despite comparable coding content for the *Trypanosoma* *brucei* and *cruzi* group trypanosomes, the latter have larger Rab, TBC and SNARE repertoires, suggesting further secondary loss in the former group. Even *Leishmania* spp. generally have slightly larger numbers of these proteins, despite smaller predicted coding content. Overall, this suggests adaptive shaping of trafficking system gene repertoires rather than changes simply reflecting genome size.

### Anterograde trafficking genes are conserved across kinetoplastids

**Fig. 5.**
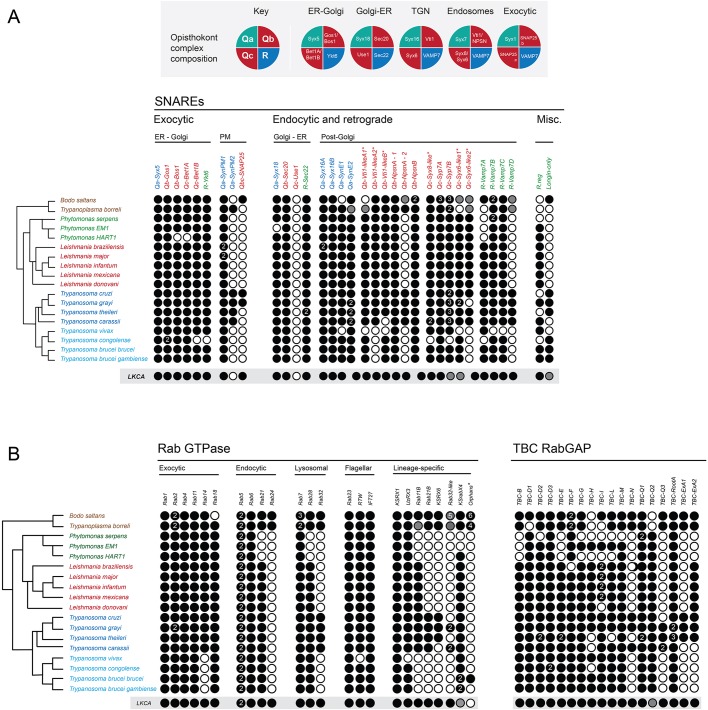
**Representation of SNAREs, Rabs and RabGAPs in kinetoplastids.** Individual subtype clades as found in kinetoplastids (assigned to known eukaryotic sub-types or lineage-specific) are shown by columns, with taxa shown as rows, with the hypothetical last common kinetoplastid ancestor as the lowest row. A schematic taxon phylogeny is at the left. Black indicates at least one member of the clade was found with phylogenetic support of 80/50/0.5 (MrBayes/PhyML-BS/PhyML-aLRT, respectively) or more; grey indicates lower support but above 50 (MrBayes) or in the case of the LKCA, indeterminable presence from given data. SNARE subtypes are shown in Fig. 5A and are colour-coded as Qa, blue; Qb, Qc, Qbc, red; and R, green/teal. Subtypes with single asterisks indicate that assignment was via BLAST only due to unresolved phylogeny. Rab and RabGAPs are shown in Fig. 5B.

The gene complements for SNAREs and Rabs at the kinetoplastid early secretory pathway are highly conserved. In opisthokonts, the SNARE complex of Qa-Syx5, Qb-Bos1, Qc-Bet1 and R-Sec22 mediates endoplasmic reticulum (ER) to Golgi and intra-Golgi transport (Hardwick and Pelham, 1992; Newman et al., 1990). A second Syx5 complex, constituting Qa-Syx5, Qb-Gos1, Qc-Sft1 and R-Ykt6 is thought to exclusively mediate COPI transport within the Golgi in animals and fungi (Ballensiefen et al., 1998). Whereas Sft1 and Bet1 are two alternate Qc SNAREs acting at early anterograde steps (Kloepper et al., 2007), they were unresolved in our phylogenies (Fig. S2B). It is likely that Bet1 fulfils the requirements of the kinetoplastid early anterograde pathway as two Bet1 paralogs are present, similar to in *H. sapiens*. Significantly *A. thaliana* has an expanded set of four of these proteins (designated as Bet11, Bet12, Sft11 and Sft12) and *D. discoidium* just one. The other SNAREs in these complexes are all singletons in kinetoplastids except for some species-specific losses (Fig. 5A) and duplication of Gos1 in *Trypanosoma* *congolense*. Syntaxin 17 cycles between the ER and the ER–Golgi intermediate compartment (ERGIC) in mammalian cells (Itakura and Mizushima, 2013) and acts in autophagosome formation and mitochondrial dynamics (Arasaki et al., 2015; Itakura et al., 2012). Syntaxin 17 is generally patchily distributed across eukaryotes and absent from all kinetoplastids, signifying a loss since divergence from the Heterolobosea. Overall, these data suggest possible differentiation in early secretory events, consistent with multiple budding pathways at the trypanosome ER, but also indicating lineage-specific evolution of these steps (Sevova and Bangs, 2009).

The retrograde pathway from the Golgi to the ER is mediated by a complex of Qa-Syx18, Qb-Sec20, Qc-Use1 and R-Sec22 in yeast (Dilcher et al., 2003). Except Use1, all are conserved across kinetoplastids, bar losses of Syx18 or Sec20 in *Phytomonas EM1* and *HART1* respectively, and duplication of Sec22 in *Trypanosoma* *theileri* (Fig. 5A). This suggests that canonical retrograde transport systems are present, consistent with the presence of a KDEL receptor and ER-retrieval signals on major ER proteins (Bangs et al., 1996; Schwartz et al., 2013).

In opisthokonts, the late exocytic SNARE complex is a specific ternary complex composed of a plasma membrane syntaxin (denoted SynPM), SNAP25 and Syb1. A SynPM is identified across eukaryotes and is likely to be ancient (Dacks and Doolittle, 2002). SynPM has a complex history in kinetoplastids, with two clear paralogs, SynPM1 and SynPM2. *Leishmania* *braziliensis* and *major* possess two copies of SynPM1 which are closely related lineage-specific duplications. The bodonid *Trypanosoma* *borreli* and *cruzi* group possess a second SynPM2 protein. No transmembrane domain (TMD) was detected in SynPM2 of the duplicated SynPM1 genes in *L. braziliensis* and *L. major*, which are otherwise identical, but for the lack of a TMD in one paralog. The TMD is also lost from the single SynPM1 in *Leishmania* *infantum* and *mexicana* but retained by *L. donovani.* The localisation of *T. brucei* SynPM1 (TbSynPM1) is similar to the TMD-containing SynPM1 from *L. major*, which localises close to the flagellar pocket, while as expected the TMD-lacking SynPM1 in *L. major* is cytosolic (Besteiro et al., 2006). Overall, this pattern indicates a considerable level of species-specific SynPM paralogs, suggesting a common requirement to differentiate this pathway.

**Fig. 6.**
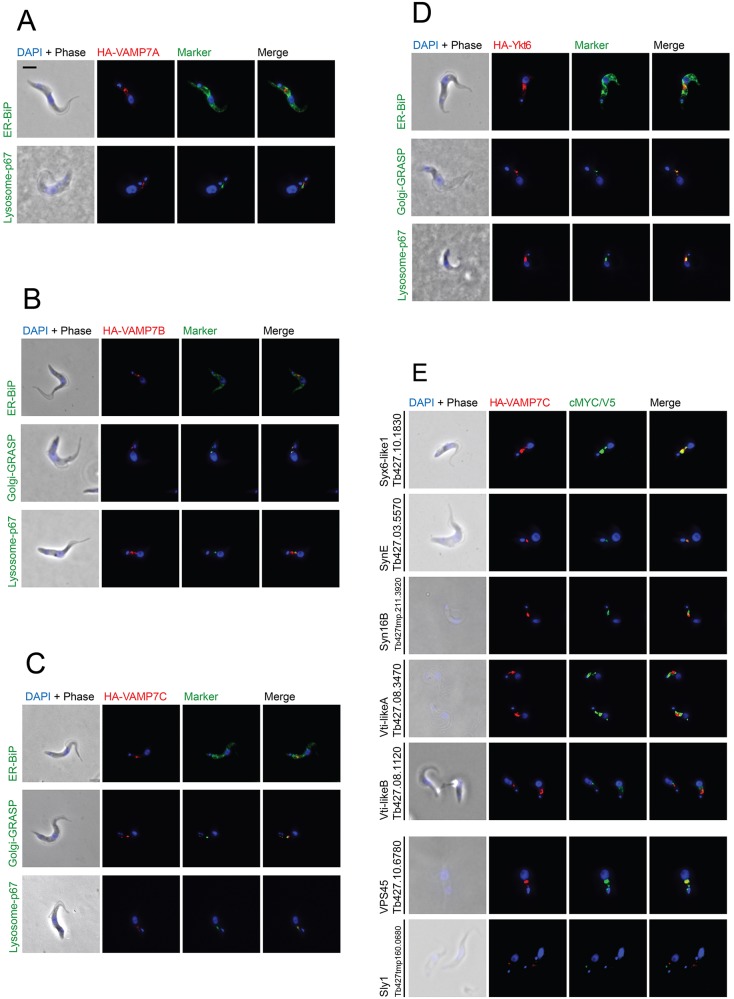
**Sub-cellular localisation of *T. brucei* R-SNAREs.** (A–D) Myc-tagged TbVAMP7A (A), HA-tagged TbVAMP7B (B), TbVAMP7C (C) and TbYkt6 (D) in procyclic *T. brucei* cells are shown. Localisation of each SNARE (red) is shown relative to markers (green) for the endoplasmic reticulum (TbBiP) (top), the lysosome (p67) (middle) and the Golgi complex (TbGRASP) (bottom). The nucleus and kinetoplast are stained with DAPI and pseudocoloured in blue. Tags were visualised with rat anti-Myc antibody or rat anti-HA antibody as appropriate. (E) Localisation of candidate TbVAMP7C interactors. HA-tagged VAMP7C (red), is shown relative to putative interactors tagged with Myc (top five rows) and V5 (lower two rows), in green. The nucleus and kinetoplast were stained with DAPI. VAMP7C was stained with rat anti-HA, SNARE interactors were stained with mouse anti-Myc antibody and non-SNARE interactors were stained with mouse anti-V5 antibody. Scale bar: 2 µm.

Putative Qbc-type SNAREs are present in *B. saltans*, *T. cruzi* and *T. grayi*, but do not cluster with canonical yeast or human Qbc SNAREs, but instead with SAR-CCTH and Excavate sequences (Fig. S2E). The eukaryotic phylogeny of synaptobrevins (Fig. S2D) is also complex, and the presence of brevins in different clusters of diverse taxa indicates that they likely emerged independently in several lineages by loss of the longin domain from a ‘VAMP7’. Moreover, unlike metazoan brevins, which are truncated at the SNARE domain, longin-less VAMP7 orthologues have an extended N-terminal domain in Apicomplexa and Euglenozoa, indicating a separate origin. A single independently evolved VAMP7 with a divergent/undetected longin domain is present in kinetoplastids, and in *T. brucei* this protein TbVAMP7C has an endosomal localisation in juxtaposition to Golgi and lysosomal markers (Fig. 6C) rather than the cell membrane.

Rab1, Rab8 and Rab18 are present in the predicted LECA cohort and associated with anterograde pathways (Elias et al., 2012). Interestingly, Rab8 is lost from all kinetoplastids, suggesting differences in post-Golgi trafficking and targeting of material to the flagellum/recycling endosome (Huber et al., 1993; Klöpper et al., 2012). Rab1 and Rab18 have a complex history, and paralogous expansion of Rab1 or Rab18 may have given rise to two lineage-specific Rabs, KSRX1 and UzRX3, which appear across the Euglenids as tandem genes and are also present in the heterolobosids; however, it may be significant that in *T. brucei* these proteins have no obvious role in trafficking (Natesan et al., 2009). While UzRX3 localisation is similar to Rab1 (early secretory pathway), KSRX1 has a more diffuse localisation (Natesan et al., 2009). Interestingly, *B. saltans* has two additional relatives of the Rab1 and Rab18 clade, suggesting further diversification of these pathways.

Post-Golgi pathway Rabs include Rab4, Rab14 and Rab11, which are mostly well-conserved, with the exception that Rab14 is lost by the *T. brucei* group (Fig. 5B). This suggests possible further adaptations of endocytic pathways, an important mechanism for immune evasion in mammalian infective African trypanosomes. Like AP-2, however (Manna et al., 2013), Rab14 is present in the other extracellular trypanosomes of the *T. cruzi* group (Fig. 5B, Fig. S3E). We also identified a Rab11-like protein (Rab11B) that likely arose from duplication of Rab11 at the base of the kinetoplastids, but this is only retained by *B. saltans*, *Leishmania* spp. and *T. cruzi* group (Fig. 5B; Fig. S3E). Rab11B may provide additional flexibility in surface remodelling, and importantly *T. cruzi* Rab11 localises to the contractile vacuole, regulating trafficking of *trans*-sialidase to the plasma membrane (Niyogi et al., 2014). Hence, kinetoplastid Rab11 and Rab11B may have distinct functions, as noted for Rab11 paralogs in mammals and Archaeplastida (Lapierre et al., 2003; Petrželková and Eliáš, 2014).

### Endocytic, retrograde and lysosomal pathway trafficking

We identified four Qa SNARE proteins (SynE1 and SynE2, Syx16A and SyxB), four Qb SNARE (VTI1-like A, B and NPSNA and NPSNB), four Qc SNAREs (Syp7A, Syp7B, Syx6-like and Syx8-like), and four R-SNAREs (VAMP7A, B, C and D) as orthologues of SNARE proteins involved in endosomal, lysosomal and retrograde trafficking pathways. For several, multiple paralogs exist, particularly in the basal bodonids and the *T. cruzi* group (Fig. 5A; Fig. S3A–D).

Except for *B. saltans* and *T. vivax*, SynE1 is present in all kinetoplastids and possibly duplicated in the parasitic bodonid *T. borreli*. SynE2 is also present in all kinetoplastids and duplicated in the *T. cruzi* group. Two paralogs of Syx16, Syx16A and Syx16B, are conserved across kinetoplastids. Thus, both SynE and Syn16 underwent duplication at least once early in the lineage evolution. As in the SynPM proteins, all Syx16A proteins lack a TMD, while all Syx16B except *T. borreli* retain the TMD. *L. braziliensis* also shows lineage-specific duplication of Syx16A, a surprisingly conserved isoform, which suggests that it must retain a function, and is perhaps a regulatory protein. Paralogous Syx16 sequences from other organisms all retain their TMD, suggesting loss of the TMD is kinetoplastid-restricted. Notably, the Sec1/Munc-like protein (SM) Vps45, which regulates Syn16, is also duplicated at the base of the kinetoplastids (Koumandou et al., 2007), and possibly in the last kinetoplastid common ancestor (LKCA), suggesting independent regulation of multiple Syn16 complexes.

The Qb (two VTI1 and two NPSN proteins), Qc (two Syp7 and two Syx6-like proteins) and R-SNAREs (four VAMP7 proteins) are expanded in kinetoplastids. Specifically, Qb-NPSNA-2, Qc-Syp7B, R-VAMP7D together with Qa-SynPM2 appear to have arisen at the base of the kinetoplastids but are retained only in the bodonid and *T.* *cruzi* groups (Fig. 5A). These SNAREs are implicated in plasma membrane trafficking in plants, suggesting they may mediate a similar pathway in the kinetoplastida. In addition, the Syp7B clade also shows evidence for bodonid and *T.* *cruzi* group-specific expansions. Most SNARE losses are scattered with no obvious pattern, but there are clear instances of co-evolutionary loss of components of predicted SNARE complexes. SNAREs Qa-SynPM2, Qbc-SNAP25 and R-VAMP7D, which could form a putative exocytic complex are all lost in African trypanosomes as well as the *Leishmania* and Phytomonad clades. Qb-VTI1-like A2, Qb-NPSNA2, and Qc-Syx6-like 2, predicted to form complexes with VAMP7, are coincidentally lost (see Fig. 5A). Except for SNAP-25 (which shows a broader pattern of loss), these are all SNAREs derived from kinetoplastid-specific expansions, and likely indicate loss of kinetoplastid-specific post-Golgi pathways.

Of the endocytic Rabs (Rab5, Rab20 or Rab24, Rab21, Rab22 and Rab50), kinetoplastids retain Rab5, Rab21 and Rab24, albeit with Rab24 bodonid restricted (Fig. 5). Early endocytic Rab5 experienced a single basal duplication in kinetoplastids and both paralogs are stably retained (Fig. S3E) and have acquired distinct functions (Pal et al., 2002). This contrasts with the fungal Ypt5, which is less well retained, with several instances of species or clade-specific expansions and losses (Pereira-Leal, 2008). Rab21 is generally stable with no major expansion in eukaryotes, but there are at least two paralogs at the base of the kinetoplastids (Fig. S3E); Rab21 mediates intermediate endosomal trafficking in *T. brucei*, a conserved function with higher eukaryotes (Ali et al., 2014). An expanded Rab21 subfamily is only retained by bodonids and the *T. cruzi* group, suggesting additional endosomal pathways. In *Phytomonas* spp., which have lost Rab21, there is clear minimisation of endocytic Rabs. Rab2 and Rab6, which mainly mediate retrograde trafficking at the Golgi complex, are also stably retained. Two copies of Rab2 are present in the bodonids and *T. grayi*, but cluster separately from the canonical Rab2.

Rab7, which mediates late endocytic trafficking, is represented by a single paralog in trypanosomatids; however, a Rab7-like protein is found in *B. saltans* and *T. borreli* (Fig. 5). Rab28 also has an endosomal function in *T. brucei* (Lumb et al., 2011) and except for Phytomonads is present across all kinetoplastids*.* Rab23 and the Rab-like intraflagellar transport protein 27 (IFT27), which are involved in the biogenesis of cilia/flagella, are both fully retained as expected. Rab32 is involved in the biogenesis of lysosome-related organelles (LROs) and autophagosome formation in mammalian cells (Hirota and Tanaka, 2009), as well as mitochondrial dynamics (Bui et al., 2010; Ortiz-Sandoval et al., 2014). In *T. cruzi*, Rab32 is involved in the biogenesis and maintenance of acidocalcisomes (Niyogi et al., 2015), and presents complex evolution in kinetoplastids, being present only in the bodonids and *T. cruzi* group, where one copy of the canonical Rab32 appears to have undergone duplication giving rise to Rab32-like proteins (Fig. 5B). Many trypanosomes may thus have reduced or alternative pathways for autophagosome biogenesis and/or mitochondrial dynamics, but by contrast, the emergence of Rab32-like proteins likely suggests the development of novel or more complex LROs, particularly in *B. saltans*, which has up to five Rab32-like proteins. TBCs associated with endosomal Rabs include TBC-B, which acts on Rab2, Rab7, Rab11 and Rab21 and TBC-D, which is a GAP for Rab1, Rab7, Rab11 and RabL5 (Fukuda, 2011; Gabernet-Castello et al., 2013). This latter TBC is implicated in recycling of VAMP7 vesicles and endosome to Golgi transport. TBC-B is retained in all kinetoplastids as a single copy but lost in the phytomonads. TBC-D is stably retained across all eukaryotes with occasional duplications, but has multiple paralogs in kinetoplastids (Fig. 5B). Other TBCs exhibit lineage-specific duplications or losses but there is no obvious co-evolutionary relationship with the Rabs. For example, TBC-F is duplicated in bodonids, four of the five *Leishmania* spp. have duplicated TBC-I (*L. donovani* is missing TBC-I), and *T. grayi* and *T. theileri* have two copies of TBC-RootA. Major TBC losses are present in *Phytomonas* spp., and all lack TBC-B and TBC-E; *P.* *serpens* and *HART1* also lack TBC-G, TBC-H, TBC-I and TBC-L, while *P. serpens* is specifically missing TBC-M. These Phytomonad TBC families are small when considered relative to the modest Rab complement.

### Orphan and unassigned SNAREs, Rabs and TBCs

Several sequences could not be unambiguously assigned. Examples include lineage-specific KSRabX4, likely a result of a *T. brucei-*specific duplication: only *T. brucei* and *T. gambiense* have two neighbouring copies with ∼52% identity, while remaining species possess one paralog (Fig. S3E). KSRabX6 is only found in the bodonids and *T. cruzi* group and BLAST searches into opistokhonts suggest similarity to Rab5 (Fig. 5B; Fig. S3E). Several other unassigned Rabs are also present in *B. saltans* and *T. borreli*. Among SNAREs, a tomosyn-like regulatory R-SNARE is present (Fig. S2D) and exhibits patchy distribution, while an unconventional longin-domain protein with no apparent SNARE domain is patchily distributed. Its structure is reminiscent of plant phytolongins, which are derived from VAMP7 (Vedovato et al., 2009) but the kinetoplastid protein appears to be closely related to another R-SNARE, Sec22 (Fig. S2E). Much of the expansion of lineage-specific paralogs is due to duplication at the base of the lineage and asymmetrical retention, highly suggestive of a genome duplication event at the kinetoplastid root.

### Conserved localisation of R-SNAREs

We are, in essence, using phylogeny and orthology as a predictor of function, which over the 10^9^ years divergence between kinetoplastids and opisthokonts, is not necessarily valid. For example, associations between orthologous SNAREs within specific complexes could have changed over such a great period. To address this directly, we tagged VAMP7A, VAMP7B and VAMP7C at the C-terminus and Ykt6 at the N-terminus to facilitate overexpression in procyclic form *T. brucei* (Fig. 6A–D). All three VAMP7s are likely largely endosomal. VAMP7A and VAMP7C are juxtaposed to the lysosome, with VAMP7A showing occasional overlap and VAMP7B being slightly more distal from the lysosome. Both VAMP7B and VAMP7C are juxtaposed to the Golgi and appear to be associated with the Golgi during its duplication (seen as two GRASP-stained puncta during cell division). As material building the new Golgi is derived from the old Golgi stack (Wang and Seemann, 2011), VAMP7B and VAMP7C may play a role in this transfer given their presence with both the Golgi stacks. The Golgi-SNARE Ykt6 does colocalise with the Golgi. These localisations are consistent with the behaviour of the yeast and human orthologues.

### Conservation of VAMP7C interactions

To examine VAMP7C interactions, we used immunoisolation and mass spectrometry. In opisthokonts, VAMP7 forms two complexes, the first with SynE, Syx8 and VTI1B, to mediate lysosomal transport, and the second with Syx6, Syx16 and VTI1A for endosomal trafficking (Jahn and Scheller, 2006). In *T. brucei*, we consistently identified VTI1-like A and B, Syx8-like, Syx6-like1, Syx16B and SynE (Table 2), a complex composition that is also consistent with their localisation (Fig. 6E). Opisthokont VAMP7 is also implicated in plasma membrane trafficking, with Qa-SynPM and Qbc SNARE SNAP-25 or SNAP-23 (Jahn and Scheller, 2006) and in plants in complex with Qa-SynPM, Qb-NPSN and Qc-Syp7 (Suwastika et al., 2008; Zheng et al., 2002). We reproducibly recovered TbNPSNA as a candidate VAMP7C interactor, albeit with poor emPAI support, but not TbSyp7, suggesting that a different *T. brucei* VAMP7 (TbVAMP7A or B) is required or that the SNARE complex composition for plasma membrane targeting is divergent. Therefore, endosomal interactions between VAMP7 and partners are apparently conserved with the opisthokont SNARE complexes.

**Table 2.**
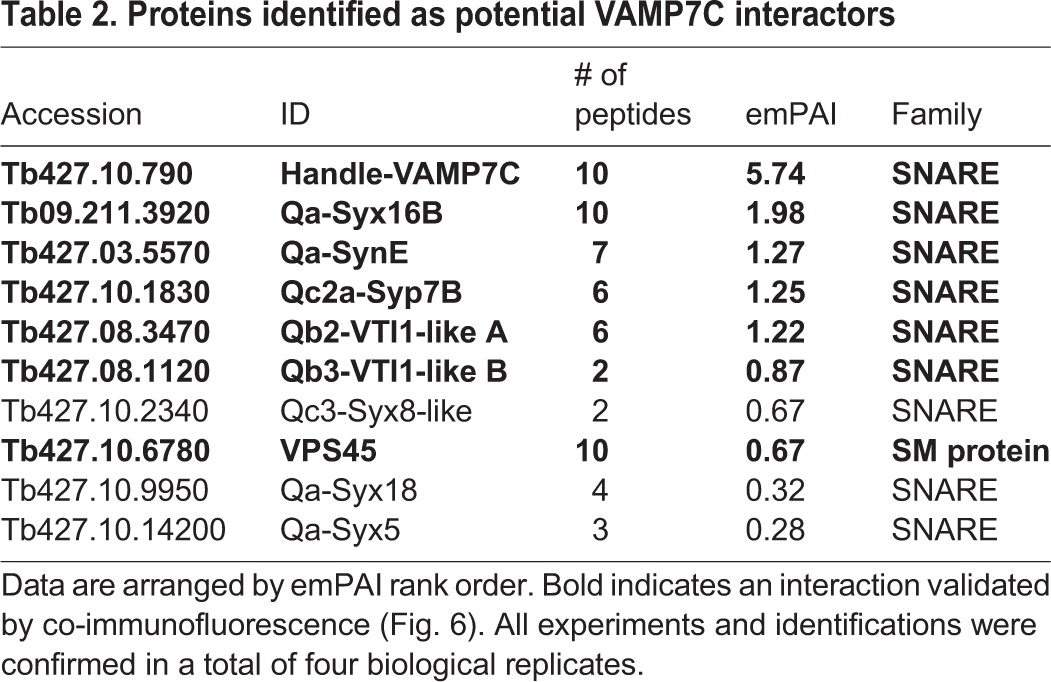
**Proteins identified as potential VAMP7C interactors**

## DISCUSSION

The kinetoplastids encompass many parasitic species with a particularly broad range of niches and life cycles. Surface components are critical to their success, which is partly reflected within the trafficking system, and our analysis here provides a comprehensive view of these processes. The repertoire of Rabs, RabGAPs and SNAREs at the LCKA resembles that predicted for LECA, and the free-living *B. saltans* possesses the largest repertoire, with several kinetoplastid-specific members that were likely to originate in the LCKA. Significantly, we also find that the frequency of paralogous pairs is high in kinetoplastids, which may indicate a whole genome duplication event at the base of the kinetoplastid lineage. Rather than a precipitous decrease as kinetoplastids transitioned from free-living to parasitic forms, there is gradual loss of trafficking genes, and likely corresponding pathway simplification (Jackson et al., 2015). Whereas some LECA proteins (e.g. SNAP-25, Rab24, Rab32 and TBC-N) are lost, a large proportion of losses are of lineage-specific duplications originating in the last common excavate ancestor or in the LCKA. Hence, radical intracellular remodelling did not accompany the transition to parasitism. The extensive bodonid repertoire is mainly due to retention of the LECA/LKCA gene complement, and the most prominent expansions appear to be Syp7 and Rab32.

The putative common ancestor of African trypanosomes seems to have had a single instance of coordinated loss of several SNARE complex subunits, specifically post-Golgi SNAREs, which is accompanied by the loss of Rabs predicted to act in similar pathways (phagocytic Rab14 and putative recycling Rab11B). *Leishmania* and trypanosomes have both lost the endosomal Rab32, Rab21B and Rab21C, SNAREs NPSN1 and SNAP25, and TBC-ExA and TBC-N. The *T. cruzi* group exhibit the greatest degree of inter-lineage variation, perhaps reflecting the varied lifestyles and disparate hosts and vectors that this group enjoys. Moreover, bodonids and the *T. cruzi* group have several clade and species-specific gains and losses indicating highly dynamic shaping of trafficking genes in these organisms.

## MATERIALS AND METHODS

### Sequence data collection

To identify SNARE sequences, a validated dataset of 26 predicted SNARE sequences obtained for *T. brucei* (Murungi et al., 2014) and 27 sequences from *L. major* (Besteiro et al., 2006) were used to query predicted proteomes for *Trypanosoma brucei brucei 927*, *T. b. gambiense*,* T. congolense*,* T. vivax*,* T. cruzi*,* T. grayi*,* Leishmania major*,* L. mexicana*, *L. braziliensis*, *L. infantum*, *Bodo saltans*, *Phytomonas serpens*, *Phytomonas EM1*, *Phytomonas HART1* (http://www.genedb.org/ or TriTrypDB) and the heterolobosid *Naegleria gruberi* (Fritz-Laylin et al., 2010) (http://genome.jgi.doe.gov/Naegr1) by BLAST. tBLASTp was used to search transcriptome data from *T. theileri*, *T. carassii*, *Trypanoplasma borrelli* and *Euglena gracilis* (S.K. and M.C.F., unpublished data). All sequences returned with e-values <10^−3^ were retained. This dataset was parsed to remove redundancies using >99% sequence identity as criteria for exclusion. ClustalW (Thompson et al., 2002) was used to align each dataset and generate neighbour-joining (NJ) trees (Saitou and Nei, 1987). Sequences which were excluded or weakly clustering were validated for a SNARE domain via Interpro (Hunter et al., 2009, http://www.ebi.ac.uk/interpro) and SNARE-DB (Kloepper et al., 2007, http://bioinformatics.mpibpc.mpg.de/snare/index.jsp). Sequences incorporating a SNARE-domain (SNARE, t/v-SNARE, sec20, syntaxin, longin and synaptobrevin) were retained. Sequences in which no domain was detected but were between 70 and 500 residues were also retained.

A pfam Ras domain (PF00071) HMMSCAN (Eddy, 1998) (http://hmmer.org) search was conducted against 50 eukaryotic proteomes, including those listed above to identify Rabs. Sequences with e-values <10^−3^ were retained and used to generate a NJ tree. Kinetoplastid Rab candidates were identified and tentatively assigned on the basis of association with defined Rab sequences. One sequence from each cluster was used as a query to define the cluster by reciprocal best hit (rbh) BLAST and another round of rbhBLAST performed using sequences from the first round as queries. Rbh requires that potential positive hits retrieve the original query when used to search. The tree was annotated accordingly to verify initial assignments. Additionally, assignments were made using Rabifier (Diekmann et al., 2011). All collected kinetoplastid sequences were classed as either a tentative Rab subfamily member or as stray. Identical procedures were undertaken with the TBC domain (pfam PF00566) and HMMSCAN to recover sequences.

For analyses addressing the evolution of NPSN and Syp7, a broader eukaryotic sampling and separate phylogenetic methods were used. Homology searches were performed using *Homo sapiens*, *Saccharomyces cerevisiae* and *Arabidopsis thaliana* sequences; protein sequences from a broad sampling of eukaryotes were retained. Positive BLAST hits for each protein of interest were aligned using MUSCLE v3.8.31 (Edgar, 2004). Where very similar sequences were present from the same or closely related organisms, those that aligned least well were removed to limit overrepresentation of some taxonomic groups in the Hidden Markov Model (HMM). For each potential orthologue identified by HMMER (e-value 0.05), reverse BLAST searches were performed as before. Homology search results were summarised using Coulson Plot Generator (Field et al., 2013).

### Phylogenetic reconstruction

SNARE, Rab and RabGAP datasets were aligned using MAFFT (Katoh et al., 2005) using the E-INS-i strategy and manually edited in Jalview (Waterhouse et al., 2009). The alignment was used to generate maximum-likelihood trees in PhyML v3.0 (Guindon et al., 2010) using the LG model with the following parameters: number of substitution rates, 4; starting tree, BioNJ, tree topology search; NNI moves and statistics, aLRT SH-like and bootstrap (100 or 1000 replicates as indicated in the figures). Bayesian inference was implemented in MrBayes v3.2 on the CIPRES server (Miller et al., 2010; Ronquist and Huelsenbeck, 2003), generally with 8×10^6^ Markov chain Monte Carlo (MCMC) generations where convergence was achieved, as measured by a splits frequency below 0.01. Substitution models employed for inferring trees were selected using ProtTest v3 (Abascal et al., 2005) for PhyML and the mixed model for MrBayes. One representative from each well supported clade (>0.9, 90, MrBayes, PhyML support respectively) along with a panel of known eukaryotic SNAREs, Rabs and TBC Rab-GAPs were analysed to determine orthology and define subfamilies. A SNARE panel was created from SNARE sequences from *Homo sapiens*,* Saccharomyces cerevisiae*,* Arabidopsis thaliana*,* Phytopthora sojae* and *Entamoeba histolytica* obtained from SNARE-DB*.* For Qb and Qc, LECA complement datasets were assembled from the searches. Alignments generated for HMMs or phylogenetic analysis were constructed using MUSCLE v3.8.31 (Edgar, 2004) and edited using Mesquite (Maddison and Maddison, 2015). Maximum likelihood phylogenies were constructed using RAxML (Stamatakis, 2014) with the Protein GAMMA model for rate heterogeneity and the LG4X substitution matrix. Values from 100 bootstraps were mapped onto the best of 20 topologies constructed from the original alignment. Bayesian phylogenetic analysis was performed using MrBayes, and run for 8–10 million MCMC generations with the Mixed substitution model, and the final average standard deviation of split frequencies decreased to less than 0.008. Several trees were constructed iteratively for each dataset, with removal of manually selected sequences to improve resolution.

### Trypanosome cell culture

Procyclic culture form (PCF) *T. b. brucei* Lister 427 were grown as previously described (Brun et al., 1979). Expression of plasmid constructs was maintained using antibiotic selection at the following concentrations: G_418_ or hygromycinB at 25 μg/ml, blasticidin at 10 μg/ml and puromycin at 2 μg/ml.

### Expression constructs

Putative trypanosome SNAREs TbVAMP7C (Tb427.10.790), TbVAMP7A (Tb427.2.5120), TbVAMP7B (Tb427.5.3560), TbYkt6 (Tb927.9.14080) were amplified from *T. b. brucei* 427 genomic DNA using Hercules DNA polymerase (Aligent Technologies).

For hemagglutinin (HA)-tag fusion, the PCR products containing sequence for a C-terminal HA-epitope were cloned into the PCF expression vector pLew79 (Wirtz et al., 1999) using AvrII and BamHI (TbVAMP7 isoforms) using the following primers: 5′-TTGTGTCCTAGGATGCTTATATCTGCCTCCTT-3′ pLewVAMP7A_F and ACTCAAGGATCCTTAAGCGTAATCTGGAACATCGTATGGGTACTTTTTGCACTTGAGGTTAG-3′ pLewVAMP7A_R; 5′-TTGTGTCCTAGGATGCCCATTAAATATAGTTG-3′ pLewVAMP7B_F and 5′-ACTCAAGGATCCTTAAGCGTAATCTGGAACATCGTATGGGTATGACTTGCAGTTGGAAAAGT-3′ pLewVAMP7B_R; 5′-TTGTGTCCTAGGATGCAGGGAGGAACAAAAAT-3′ pLewVAMP7C_F and 5′-ACTCAAGGATCCTTAAGCGTAATCTGGAACATCGTATGGGTACTTCTTTTCCTCTTTTTTAC-3′ pLewVAMP7C_R.

The PCR product of TbYkt6 was cloned into pHD1034 containing an N-terminal HA-epitope using HindIII and AflI using the following primers: 5′-GGCCAAGCTTTATACTCCCTGGCAAT-3′ pHD1034Ykt6F and 5′-CCGTCTTAAGTCACATGACGGTGCAACA-3′ pHD1034Ykt6R.

Putative SNARE interactors TbSyx16B (Tb427tmp.211.3920), TbSynE (Tb427.03.5570), TbVTI1-likeA (Tb427.8.3470), TbVTI1-likeB (Tb427.08.1120), TbSyx6-like1 (Tb427.10.1830) and TbSyx8-like (Tb427.10.2340) were also similarly amplified. For 6× Myc tagging, the product of each gene was cloned into pRPΔOP (with thanks to Lucy Glover, Institut Pasteur, Paris, France) containing 6× cMyc-epitope using HindIII and XbaI using the following primers: 5′-GCGCGCAAGCTTATGAGCGGGGACGGCGTTGG-3′ pRPCTb427.8.1120F and 5′-GCGCGCTCTAGAAACTTTCCCCAGAAACTTCC-3′ pRPCTb427.8.1120R; 5′-GCGCGCAAGCTTATGGACGATCCAAGTTGGCA-3′ pRPCTb427.3.5570F and 5′-GCGCGCTCTAGATACTTTATGGTACGCAACGA-3′ pRPCTb427.3.5570R; 5′-GCGCGCAAGCTTATGTCGTCTCTGCAAGATCC-3′ pRPCTb427.10.1830F and 5′-GCGCGCTCTAGAACTAAAGACACAATAGAAGA-3′ pRPCTb427.10.1830R; 5′-GCGCGCAAGCTTATGTCTAAACAAGAA-3′ 2F_PRP_Tb427.10.2340_C and 5′-GCGCGCTCTAGAAAGTATTAAAAGCAC-3′ 2R_PRP_Tb427.10.2340_C; 5′-GCGCGCAAGCTTATGTCATCTGATCTT-3′ 3F_PRP_Tb427.08.3470_C and 5′-GCGCGCTCTAGACTTCCAAAATACAAT-3′ 3R_PRP_Tb427.08.3470_C; 5′-GCGCGCAAGCTTATGGCGACCCGTGAC-3′ 4F_PRP_Tb427.211.3920_C and 5′-GCGCGCTCTAGAAGACAGCATCTTTTG-3′ 4R_PRP_Tb427.211.3920_C.

Putative interactors TbVps45 (Tb427.10.6780) and TbSly1 (Tb427tmp.160.0680) were cloned into pMOT vector (Oberholzer et al., 2006) with 3xV5 tag using the following primers: 5′-AGGTCCTGTGCACGCCTGCATCGGTGGGACTGGAGTCCTTAACAGTGAAACCTTCCTGAGCCTGCTAGCAGCGCACGCAGGTACCGGGCCCCCCCTCGAG-3′ VPS45pMOT_F and 5′-GTATTTTGGTTTCGTTTATTCATACCACCATGCGGAGGCGCAATGTCCCCGCCAAAACAGGCGAGGGCGGCACATGGCGGCCGCTCTAGAACTAGTGGAT-3′ VPS45_pMOT_R; 5′-GGTTAGTTATGGCTGTACCGCAATGCTGACGGGGAATGAAGCACTGCGCCAGCTTACTGTTCTTGGTGAAGGAATATCAGGTACCGGGCCCCCCCTCGAG-3′ Sly1_pMOT_F and 5′-AAAGCACGTTAGGATAGTATCTGAAAGTGGGAAAACGCCAAATGGCACAAAGACCAAAACGGCCGGGCCGGTGCTGGCGGCCGCTCTAGAACTAGTGGAT-3′ Sly1_pMOT_R.

All constructs were verified by sequencing and linearised with NotI prior to transfection into cells. Clonal transformants were selected by resistance to antibiotics as relevant to each vector and cell line.

### Transfection of PCF *T. brucei*

1.6×10^7^ cells per transfection were harvested at 4°C, washed in cytomix and resuspended in 500 μl cytomix. Electroporation was performed with 5–15 μg of linearised DNA using a Bio-Rad Gene Pulser II (1.5 kV and 25 μF). Cells were transferred to 9.5 ml SDM-79 medium and incubated for 6 h, after which selection antibiotics were added. The cells were then diluted into 96-well microtiter plates. Positive transformants were picked into fresh selective medium 10–15 days post transfection.

### Identification of protein–protein interactions

Interactions between VAMP7C (Tb427.10.790) and other trypanosome proteins were analysed by immunoisolation. 5×10^10^ procyclic cells habouring VAMP7C tagged at the C-terminus with HA were lysed by mechanical milling in a Retsch Planetary Ball Mill PM200 using liquid nitrogen cooling (Retsch, UK). Aliquots of powder were thawed in solubilisation buffer (50 mM Tris-HCl pH 8.0, NaCl 100 mM, 1% Triton X-100 or CHAPS+1 mM NEM, and 50 mM Tris-HCl pH 8.0, 100 mM NaCl, 5 mM EDTA, 1% Triton-X100 and/or 0.5% Triton-X114+1 mM NEM). VAMP7C::HA was isolated using Pierce anti-HA magnetic beads. All washes were in the same buffer without NEM. Following analysis of an aliquot by SDS-PAGE, affinity-isolated proteins were precipitated in 90% ethanol (v/v). The precipitated samples were trypsinised and analysed by liquid chromatography tandem mass spectrometry at the University of Dundee Fingerprints Proteomics Service. Peak lists were submitted to ProFound and searched against an in-house *T. brucei* database using data from GeneDB (www.genedb.org). An untagged wild-type cell line treated identically served as background control.

### Immunofluorescence microscopy

Cells were prepared as previously described (Leung et al., 2008). Antibodies were used at the following concentrations: rat anti-HA (cat. no. 11867423001, Roche) 1:1000; mouse anti-cMyc (Santa Cruz Biotechnology 9E10) 1:500; mouse anti-p67 (James Bangs, University of Wisconsin-Madison, WI) 1:1000; rabbit anti-GRASP (Graham Warren, Vienna, Austria) 1:500 in 20% fetal calf serum (FCS) in PBS (v/v). Wide-field epifluorescence images were acquired using a Nikon Eclipse E600 epifluorescence microscope equipped with a Hamamatsu ORCA CCD camera, and data captured using MetaMorph (Universal Imaging, Marlow, UK).

### Western blotting

Whole-cell lysates in SDS-PAGE sample buffer containing 10^7^ cells/lane were resolved by SDS-PAGE. Proteins were transferred to polyvinylidene fluoride membranes (Millipore) and blocked using 5% semi-skimmed milk. Antigens were visualised using standard methods. Primary antibody concentrations were: rat anti-HA (cat. no. SC-40, Roche) at 1:10,000, mouse anti-cMyc (cat. no. ab39688, Abcam) 1:5000. Primary antibody binding was detected using horseradish peroxidase (HRP)-conjugated anti-IgG antibodies (Sigma-Aldrich) at 1:10,000. Detection of HRP-conjugated secondary antibody was achieved with chemiluminescence and luminol.
